# Role of Bisphosphonates in Postmenopausal Women with Osteoporosis to Prevent Future Fractures: A Literature Review

**DOI:** 10.7759/cureus.5328

**Published:** 2019-08-06

**Authors:** Bashir Imam, Kashif Aziz, Mehreen Khan, Tayyaba Zubair, Amna Iqbal

**Affiliations:** 1 Internal Medicine, Jackson Park Hospital and Medical Center, Chicago, USA; 2 Neurology, Jersey Neurosciences, New Jersey, USA; 3 Internal Medicine, George Washington University School of Medicine and Health Sciences, Washington DC, USA; 4 Internal Medicine, Desai Medical Center, Ellicott City, USA; 5 Internal Medicine, Rochester General Hospital, Rochester, USA

**Keywords:** : bisphosphonate, postmenopausal osteoporosis, fracture risk reduction, side effects

## Abstract

Postmenopausal women who have osteoporosis are at increased risk of future fractures. Bisphosphonates are drugs that are used to treat osteoporosis by acting on the osteoclasts to inhibit bone resorption. Several studies have shown that bisphosphonates can maintain or even increase bone mineral density in osteoporosis patients. This review study analyzed the literature on clinical experiments with bisphosphonate therapy in postmenopausal women to determine if these drugs are efficacious in preventing future fractures. Four out of five studies found that women treated with bisphosphonates were at a decreased risk of future fractures, and six of six studies found that bisphosphonate therapy increases bone mineral density relative to placebo control. Although further work is warranted to understand the level of bone mineral density increase that is associated with fracture prevention, this study implies that bisphosphonate therapy can be used to help prevent future fractures in postmenopausal osteoporotic women. The study is significant in that it helps to underscore the efficacy of bisphosphonate therapy in postmenopausal women, and it may be generalizable to other populations with osteoporosis who are at increased risk of fractures.

## Introduction and background

Postmenopausal women are at increased risk of osteoporosis, and an estimated 40 million women in the United States are estimated to have low bone mineral density [1]. Such loss of bone mineral density and the resultant deterioration of bone architecture are known to lead to increased risk of fractures [2]. Fractures of the hip and spine are the most common that occur as a result of osteoporosis and they can lead to reduced quality of life, dependent living situations, and increased risk of death, in addition to psychological problems such as lowered self-esteem [1]. Bisphosphonates are drugs that are used to treat osteoporosis. They block the action of the bone cells known as osteoclasts and thereby inhibit the resorption of the bone itself [3]. Bisphosphonates are the first line of defense against osteoporosis and can decrease the rate of bone turnover, increase bone mineral density, and may thereby reduce the risk of fractures in patients with osteoporosis [4]. Because postmenopausal women are at increased risk of osteoporosis and resulting fractures, it stands to reason that bisphosphonates might be used in this group to prevent future fractures. The purpose of the present paper is to review the literature on therapy in postmenopausal women to determine if these drugs are efficacious in preventing future fractures. Specifically, the goal is to determine if there is clinical evidence that supports the use of bisphosphonates to reduce the risk of future fractures. Interest Hypothesis states that postmenopausal women who receive bisphosphonate treatment will have a reduced fracture risk relative to women who do not receive the treatment. The reduced fracture risk was measured by assessing the number of subsequent fractures and/or by assessing the bone mineral density following bisphosphonate therapy.

## Review

Materials and methods

A keyword search strategy was used to search for databases to locate abstracts for the study. The databases PubMed and ClinicalTrials.gov were searched using combinations of the key-words “osteoporosis,” “fracture,” “post-menopause,” and “bisphosphonate.” The parameters “human” and “English language” were selected. All bisphosphonates were included, administered either orally or intravenously (IV), according to the established protocols for administration and without co-medications, in postmenopausal women ages 65 or older who are at risk, including those with comorbidities, throughout the entire world. Inclusion criteria for journals included those that are respectable, publishing peer-reviewed articles with clearly presented methodology sections; follow-ups of more than one year and in which primary outcomes were the number of fractures of any localization and/or bone density, and secondary outcomes were side effects. All dates up to the present time were searched. Randomized clinical trials with a follow-up period of at least one year were chosen; trials without randomization or at least one-year follow-up were excluded. Studies that are not randomized controlled trials or systematic reviews of such were excluded. Studies were chosen with outcome data that included either fracture incidence after at least a one-year follow-up period, bone mineral density after at least a one-year follow-up period, or both. Secondary outcomes were the side effects. Because a limited number of articles with all of these criteria were found, and because the data were not entirely comparable across all located studies, statistical analyses were not performed. We screened the titles and abstracts to select the articles for full-text review. The articles were evaluated using the center for evidence-based medicine (CEBM) appraisal worksheets.

Results

**Figure 1:**
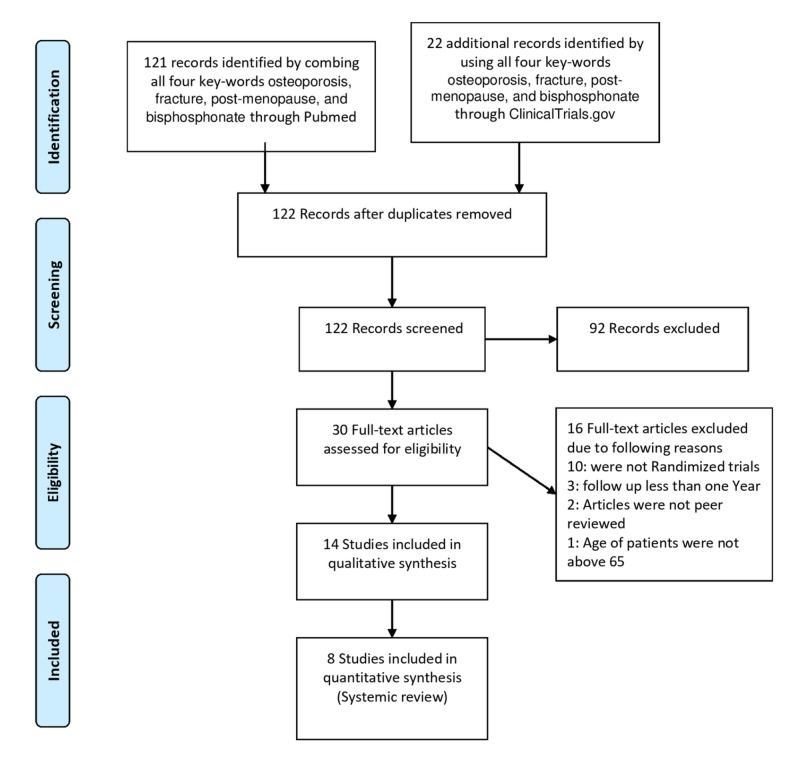
Preferred reporting items for systematic reviews and meta-analyses (PRISMA) 2009 flow diagram

Using the preferred reporting items for systematic reviews and meta-analyses (PRISMA) 2009 flow diagram, the search for combined four key-words (“osteoporosis”, “fracture”, “post-menopause”, and “bisphosphonate” resulted in 143 abstracts. Results for these 143 records were examined. After removing duplications and using inclusion criteria, there were a total of eight studies that involved postmenopausal women ages 65 or older who had osteoporosis and who were part of a randomized, placebo-controlled clinical trial. Actual PRISMA diagram is shown in Figure 1. Outcomes assessed for these trials involved fractures after at least a one year follow-up period and/or a comparison of the change in bone mineral density relative to controls. All eight of these studies were included in the final analyses. Only five of the trials reported fracture data, six of the trials reported bone mineral density data, and three of the trials reported side effects. No quantitative synthesis was performed because the reporting of the data was not consistent across all studies.

Fractures

Five studies reported data on fractures [5-9]. Osaki et al. studied 529 postmenopausal women and found that five out of 173 in the treatment group (2.89%) and 32 out of 356 in the control group (8.99%) suffered fractures. The authors reported that this was a statistically significant difference at the p=0.01 level [5]. Jacques et al. performed a study on 7736 postmenopausal women and assessed the prevalence of new vertebral fractures. They found a total of 308 fractures in 2976 women in the control group (10.4%) and a total of 23 fractures in 3195 women in the treatment group (0.7%) and reported that this was a significant difference at p<0.001 [6]. Greenspan et al. had the opposite findings, in that 17 of 89 (20%) in the treatment group and 14 of 92 (16%) in the placebo control group had vertebral fractures [7]. Together, these data indicate that the three studies assessed a total of 6881 elderly postmenopausal osteoporotic women for fracture risk after at least one year of taking bisphosphonate therapy. Two of the studies found independently of the other that fracture incidence was significantly less in the bisphosphonate treatment group than in the placebo control group, but the third found the opposite [5-7]. If the data for these studies are taken together, then 357 out of 3421 control participants suffered a fracture, and 42 out of 3460 treatment participants suffered a fracture, for incidence frequencies of 10.4 % and 1.2%, respectively. A fourth study also reported data on fracture rates; however, it did not report them per participant, but rather in fracture rates per 100 patient-years. The authors examined 66 postmenopausal osteoporotic women who took the bisphosphonate etidronate. During the period of the study from 60 to 150 weeks, the authors found that there was a statistically greater number of fractures per 100 patient-years in the placebo group (54/100 patient-years) relative to the treatment group (six per 100 patient-years). This difference was significant at the p=0.023 level [8]. A fifth study also reported on fracture data, but again, the fracture rates were in 100 patient-years units rather than by individual patients. Reid et al. examined the use of pamidronate in a two-year study of 48 postmenopausal osteoporotic women. They found that there was a trend for vertebral fracture rates to be lower in the treatment group (13/100 patient-years) relative to the placebo group (24/100 patient-years), but this was not significant at the p<0.05 level, although it was significant at the p<0.1 level (p=0.07) [9].

Bone mineral density

Six studies reported a change in bone mineral density over the study period of at least one year [7-12]. Each of these studies had a slightly different protocol for assessing change in bone mineral density in the control and treatment groups, and each of them had a somewhat different method of reporting their results, so they will not be quantitatively analyzed, but instead reported here individually. Greenspan et al. conducted a study of 181 women age 65 or older who had osteoporosis. They took either zoledronic acid or placebo in IV form, and then their hip and spine bone mineral density was assessed after 12 and 24 months. They reported that at 12 and 24 months, bone mineral density changes were greater in the treatment groups by 3.2% and 3.6% respectively for the hip, and by 1.8% and 3.6% at 12 and 24 months respectively for the spine, and all of these differences were significant at the p<0.01 level [7]. Storm et al. also examined bone mineral density among 66 postmenopausal women. They found that vertebral bone mineral density significantly increased in the treatment group, while it decreased over the study period in the placebo group. Their results indicated an overall difference of eight percentage points between the two groups at the end of the study. These results were significant at the p<0.01 level [8]. Reid et al. examined the effects of pamidronate on bone mineral density in the total body, lumbar spine, and femoral trochanter in the 48 postmenopausal women. Individuals in the treatment group at all body areas had significantly increased bone mineral density at 1.9% for the total body, 7% for the lumbar spine, and 5.4% for the femoral trochanter. There were no increases seen in bone mineral density for individuals in the placebo group [9]. Adami et al. studied the bisphosphonate alendronate sodium relative to placebo and intranasal salmon calcitonin in a two-year trial of 286 postmenopausal women with osteoporosis. They found that two different doses, 10 and 20 mg, of alendronate sodium, increased bone mineral density in the lumbar spine by 4.7% and 6.1%, respectively, relative to placebo or intranasal salmon calcitonin. They also examined the femoral neck and trochanter bone mineral density at 10 and 20 mg doses and found that bone mineral density increased for the femoral neck by 3.1% for both doses, and that trochanter density was increased by 3.3% and 3.8% respectively for the doses [10]. Grey et al. evaluated the antiresorptive effects of zoledronate in 180 postmenopausal women. They found that after two years there was a significantly greater increase in bone mineral density in the spines and hips of the treatment groups (divided into three groups with different dosages) relative to the placebo group. The changes in bone mineral density in the spines were 4.4%, 5.5% and 5.3% for 1 mg, 2.5 mg, and 5 mg zoledronate, respectively. The changes in bone mineral density in the hips were 2.6%, 4.4%, and 4.7% for 1 mg, 2.5 mg, and 5 mg zoledronate, respectively. All of the reported changes were significant at p<0.001 [11]. Popp et al. analyzed the effects of zoledronate and placebo on the bone mineral density of the spine in 107 postmenopausal women. The study was a three-year protocol, and the authors found that there were significantly greater positive changes in bone mineral density in the treatment group relative to the placebo group. At the three-year point, there was a 9.58% increase in bone mineral density of the spine in the treatment group relative to the placebo group, and this change was significant at the p<0.0001 level [12].

Side effects

Side effects/adverse events occurred in some studies [5,7,11]. Osaki et al. found that there were adverse events in 20.7% of the treatment patients and 21.1% of the control patients. The most frequent adverse events reported were gastrointestinal disorders in the treatment group and hip fracture in the control group. Other adverse events included cardiac disorders, death, pneumonia, fracture of the radius, fracture of the spine, dementia, and musculoskeletal and connective tissue disorders [5]. Greenspan et al. reported that 97% of participants in their trial had an adverse event, and 64% had a serious adverse event, but there were no statistically significant differences in adverse events between treatment and placebo groups. Side effects reported were cardiac disorders, falling, headache, pyrexia, fatigue, arthralgias, myalgias, and flu-like symptoms [7]. Grey et al. reported that one participant in the treatment group developed iritis [11]. The summarised results of the study are given below in the tabular form (Table 1).

**Table 1:** Study Results Intravenous (I.V.), Administration (Admin.)

Author(s)	Drug(s)	Admin. Mode	Fracture (location)			Bone Mineral Density (dose and/or location)			Side Effects	Risks (if addressed)
			Control	Experimental		Control	Experimental			
Osaki et al. [5]	Risedronate	Oral	8.99% (hip)	2.89% (hip)					GI disorders, fractures, cardiac disorders, death, pneumonia, dementia, musculoskeletal & connective tissue disorders	GI disorders
Jacques et al. [6]	Zoledronic acid	I.V.	10.4% (vertebral)	0.7% (vertebral)						
Greenspan et al. [7]	Zoledronic acid	I.V.	16% (vertebral)	20% (vertebral)		-/- (hip, spine)	3.6%/3.6% (hip, spine)		Cardiac disorders, falls, headache, pyrexia, fatigue, arthralgias, myalgias, flu-like	Falls
Storm et al. [8]	Etidronate	Oral	54/100 patient years (vertebral)	six per 100 patient years (vertebral)						
Reid et al [9]	Pamidronate	Oral	24/100 patient years (vertebral)	13/100 patient years (vertebral)		0/0/0 (body, spine, femur)	1.9%/7%/5.4% (body, spine, femur)			
Adami et al. [10]	Alendronate	Oral				4.7%/3.1% (10 mg/20mg)	6.1%/3.8% (10 mg/20mg)			
Grey et al. [11]	Zoledronate	I.V.				-/-/- (spine) -/-/- (hip)	4.4%/5.5%/5.3% (1mg/2.5mg/5 mg spine) 2.6%/4.4%/4.7% (1mg/2.5mg/5 mg hip)		iritis	
Popp et al [12]	Zoledronate	I.V.				-(spine)	8% (spine)			

Discussion

Overall, despite a few conflicting results, the study supports the hypothesis that bisphosphonate therapy in postmenopausal women with osteoporosis can prevent future fractures. The first type of data, based upon the randomized clinical trial literature, looked at rates of fractures in women who used bisphosphonate treatments and those who used placebo only. Five studies met all of the criteria to be included in this literature evaluation, and four of the five studies indicated unequivocally and at a statistically significant level that postmenopausal osteoporotic women who are given bisphosphonate treatment are less likely to have a future fracture than women who do not take the bisphosphonate treatment. However, one of the studies, Greenspan et al. indicated that fracture rates post-treatment was significantly greater in the treatment group, rather than in the placebo group [7]. It is interesting to think about the Greenspan et al. study in light of a three-year analysis of the bisphosphonate drugs ibandronate and risedronate among a large (N=1053) group of postmenopausal Japanese women [13]. That study found that among women who had increased bone mineral density after six months of taking either bisphosphonate had a reduced risk of fracture relative to that in women who also used the bisphosphonate treatment but did not have an increase in bone mineral density. It would be interesting to examine the Greenspan et al. data by stratifying the sample at certain bone mineral density gain parameters to see if there is a bone mineral density gain cutoff that can serve to predict fracture risk in osteoporotic postmenopausal women who have received bisphosphonate treatment.

Another outcome that was studied for this paper was bone mineral density in treatment and control groups. Because of the differences in the ways that these studies were conducted and reported, no further statistical analysis is possible for the present paper. However, each of these studies independently found that the bisphosphonates examined significantly increased the bone mineral density in the treatment groups relative to the placebo groups. All studies reviewed for this paper found that bone mineral density increased in the treatment groups relative to the control groups. If bone mineral density can be taken to indicate a reduced risk of fracture, then these studies support the role of bisphosphonate therapy in postmenopausal osteoporotic women to prevent future fractures.

Concerning the third outcome, side effects, three studies did report adverse events/side effects, and these must be taken into consideration by any potential prescriber of bisphosphonates. Patient history and risk of fractures should be weighed against the risks associated with the potential adverse events.

Both oral and intravenous treatments showed bone mineral density increases and, except for a single study, both types of administration of the therapy showed a reduction in fracture risk over time. Future research on the best bisphosphonate with the optimal regimen for treatment of osteoporosis and prevention of fracture risk in postmenopausal women is called for. Additionally, although all studies did not report side effects, three of them did, and this was only over the period of time for a few years maximum. Further work on the risks and benefits of long-term bisphosphonate therapy would be advisable.

Critical appraisals for each study

Osaki et al. was a prospective matched cohort study and did not seem to have been randomized, nor was there blinding. The matching design made the subjects similar at the beginning of the study. Only fifty-nine percent of the original subjects completed the study. Treatment effects were measured in fracture incidence over 36 months and were 4.3% in the treatment group compared to 13.1% in the control group, which was significant at p=0.10. Because this is a cohort study, it is more difficult to ascertain cause and effect in terms of the efficacy of the treatment versus the control group [5]. Jacques et al. did not report their randomization procedures in this article. The study was double-blind, and the groups were similar on demographic variables at the beginning of the study. The groups were treated equally except for the interventions. Because the study analyzed data from a different study, there were no participant drop-outs. The treatment effects were measured in bone mineral density rates and fracture rates as described above. This is a very large study, and as such, the data are likely generalizable to all postmenopausal women with osteoporosis. A criticism of this study is that it did not include data on side effects [6].

Greenspan et al. used computerized randomization accomplished by the study biostatistician. Groups were statistically similar at the beginning of the study and were treated equally except for the intervention. Seventy-six percent of the enrolled participants completed the study. The treatment effects were measured in fracture rates and bone mineral density rates as reported above. One critique of this study is that although subjects in both groups were demographically similar to each other at baseline, the treatment group had more subjects with frailty, falls, diabetes and anticonvulsant use, and this could have biased the data to some extent [7]. Storm et al. used computer-generation to randomize their patients. The study was double-blinded, and the groups were similar at the beginning of the protocol. Sixty-one percent of the enrolled participants completed the study. Treatment effects were measured as bone mass and fracture rates, as described above. This is a moderate-sized study of 66 women who were followed-up for three-years, and it is able to say that the bisphosphonate therapy increased bone mineral density in the women studied relative to the women who only had a placebo. A criticism is that this study did not include a report of side effects [8].

Reid et al. used computerized randomization of their participants. The study was double-blinded, and the subjects were similar demographically at the beginning of the protocol. There were no reported drop-outs, and 48 women participated. Treatment effects were reported in both bone mineral density changes and fracture rates, as described above. This is a moderate-sized study of 48 women, but it was continued for two years, and the measurements of the bone mineral density were taken every six months, providing good estimates of change in bone mineral density over that time period. A criticism of this study is that it did not include a report of side effects [9]. Adami et al. were unclear about their randomization procedure; however, the groups were similar to each other at the beginning of the study, and there were no statistically significant differences between them. The groups were treated equally except for the intervention. Measures were objective, and both patients and researchers were blind as to the grouping. The treatment effects were 4.7% and 6.1% for the 10 and 20 mg respective doses at the femoral neck, and 3.3% and 3.8% at the trochanter, respectively. This study has a large sample size and is generalizable to all postmenopausal women. A criticism of this study is that it did not include an assessment of side effects [10].

Grey et al. used computerized randomization accomplished by the study statistician using a variable block size schedule. There were no demographic differences in the groups at the beginning of the study, and they were treated equally except for the intervention. Ninety-three percent of enrolled subjects completed the study. The treatment effects were measured in bone mineral density rates at 4.4%, 5.5% and 5.3% for 1mg, 2.5 mg, and 5 mg zoledronate, respectively. The changes in bone mineral density in the hips were 2.6%, 4.4%, and 4.7% for 1 mg, 2.5 mg, and 5 mg zoledronate, respectively. A critique of this study is that bone mineral density was used as a surrogate for fracture risk, and it would have been helpful to have fracture incidence reported as well [11]. Popp et al. did not describe the randomization procedures in this article. The study was double-blind, and the groups were similar on demographic variables at the beginning of the protocol. The groups were treated equally except for the interventions. There were 107 patients included in the study and there appear to have been no losses to follow-up, but there were a few cases in which someone did not show up for a bone scan, according to the article. Treatment effects were measured in bone mineral density changes over three years. The authors found a 9.58% increase in bone mineral density of the spine in the treatment group relative to the placebo group, and this change was significant at the p<0.0001 level. The authors point out that although their study was large enough to compare the effects of the treatment versus the control, it was not large enough to establish the predictive value of the bone mineral density measurements in regard to fracture risk. Also, this study did not assess side effects, which is important to know for both the short- and long-term health of the women being treated [12].

## Conclusions

Postmenopausal women with osteoporosis who have suffered a fracture are at increased risk of future fractures due to loss of bone mineral density and deterioration of bone architecture. Bisphosphonates are drugs that can treat osteoporosis by inhibiting the resorption of bone, and thereby maintain or even increase bone mineral density and help to maintain the bone architecture. This review study searched the peer-reviewed scientific literature for clinical trials and/or systematic reviews and meta-analyses concerning the use of bisphosphonates to prevent future fractures in postmenopausal osteoporotic women. Four out of five studies found that women on bisphosphonate therapy were at significantly decreased risk of future fractures, although one smaller study found the opposite. Six studies found that bisphosphonate therapy significantly increased bone mineral density. The results indicate that bisphosphonate therapy can be used to increase bone mineral density and decrease the risk of future fractures, but some further research is warranted concerning the amount of bone mineral density increase necessary to reduce the risk of future fractures.
